# Supplementary material for: HIVprotI: an integrated web based platform for prediction and design of HIV proteins inhibitors
Source: J Cheminform. 2018 Mar 9;10:12. doi: 10.1186/s13321-018-0266-y (PMC5845081; doi:10.1186/s13321-018-0266-y)
Supplement: Supplementary file 2 — Additional file 2. Source code of HIVProtI web server. [file 13321_2018_266_MOESM2_ESM.zip › HIVprotI_Source-code/hivprotI/weka-3-6-6/doc/weka/classifiers/bayes/net/MarginCalculator.JunctionTreeNode.html]

MarginCalculator.JunctionTreeNode


---


|  |  |  |
| --- | --- | --- |
| **Overview** \| **Package** \| **Class** \| **Tree** \| **Deprecated** \| **Index** \| **Help** \| **Weka's home** | |  |
| **PREV CLASS**   **NEXT CLASS** | **FRAMES**    **NO FRAMES**     **All Classes** |  |
| SUMMARY: NESTED \| FIELD \| CONSTR \| METHOD | DETAIL: FIELD \| CONSTR \| METHOD |  |


---


## weka.classifiers.bayes.net Class MarginCalculator.JunctionTreeNode

```
java.lang.Object
  weka.classifiers.bayes.net.MarginCalculator.JunctionTreeNode
```

**All Implemented Interfaces:**: java.io.Serializable, RevisionHandler

**Enclosing class:**: MarginCalculator

---

``` public class MarginCalculator.JunctionTreeNode extends java.lang.Object implements java.io.Serializable, RevisionHandler ```

**See Also:**: Serialized Form

---

| **Field Summary** | |
| --- | --- |
| `java.util.Vector` | `m_children` |
| `int[]` | `m_nNodes`             nodes of the Bayes net in this junction node |


| **Method Summary** | |
| --- | --- |
| `void` | `addChildClique(MarginCalculator.JunctionTreeNode child)` |
| `java.lang.String` | `getRevision()`             Returns the revision string. |
| `void` | `initializeDown(boolean recursively)` |
| `void` | `initializeUp()` |
| `void` | `setEvidence(int nNode, int iValue)` |
| `void` | `setParentSeparator(MarginCalculator.JunctionTreeSeparator parentSeparator)` |
| `java.lang.String` | `toString()` |

**Methods inherited from class java.lang.Object**

`equals, getClass, hashCode, notify, notifyAll, wait, wait, wait`

**Field Detail**

### m\_nNodes

```
public int[] m_nNodes
```

:   nodes of the Bayes net in this junction node

---


### m\_children

```
public java.util.Vector m_children
```


**Method Detail**

### setParentSeparator

```
public void setParentSeparator(MarginCalculator.JunctionTreeSeparator parentSeparator)
```

---


### addChildClique

```
public void addChildClique(MarginCalculator.JunctionTreeNode child)
```

---


### initializeUp

```
public void initializeUp()
```

---


### initializeDown

```
public void initializeDown(boolean recursively)
```

---


### toString

```
public java.lang.String toString()
```

:   **Overrides:**: `toString` in class `java.lang.Object`

---


### setEvidence

```
public void setEvidence(int nNode,
                        int iValue)
                 throws java.lang.Exception
```

:   **Throws:**: `java.lang.Exception`

---


### getRevision

```
public java.lang.String getRevision()
```

:   Returns the revision string.

    :   **Specified by:**: `getRevision` in interface `RevisionHandler`
    :   **Returns:**: the revision


---


|  |  |  |
| --- | --- | --- |
| **Overview** \| **Package** \| **Class** \| **Tree** \| **Deprecated** \| **Index** \| **Help** \| **Weka's home** | |  |
| **PREV CLASS**   **NEXT CLASS** | **FRAMES**    **NO FRAMES**     **All Classes** |  |
| SUMMARY: NESTED \| FIELD \| CONSTR \| METHOD | DETAIL: FIELD \| CONSTR \| METHOD |  |


---
